# High miR-324-5p expression predicts unfavorable prognosis of gastric cancer and facilitates tumor progression in tumor cells

**DOI:** 10.1186/s13000-020-01063-2

**Published:** 2021-01-11

**Authors:** Zhong Zheng, Jun Li, Junyan An, Yikuan Feng, Lirong Wang

**Affiliations:** 1 Department of Gastroenterology, Weifang People’s Hospital, Weifang, 261041 Shandong China; 2 Department of General Surgery, Weifang Municipal Hospital, Weifang, 261021 Shandong China; 3 Department of Gastroenterology, Sunshine Union Hospital, No.9000 Yingqian Road, Weifang, 261031 Shandong China

**Keywords:** MicroRNA-324-5p, gastric cancer, Prognosis, Proliferation, Migration, Invasion, PTEN

## Abstract

**Background:**

Gastric cancer (GCa) is one of the six major malignancies in the world with low survival rate. Although there are advances in therapeutic approaches, the prognosis of patients with GCa remains not optimistic. Therefore, this study aimed to evaluate the prognostic value of miR-324-5p, as well as its functional role in GCa progression.

**Methods:**

The expression of miR-324-5p in tumor tissues and cell lines was examined using real-time quantitative PCR. The prognostic value of miR-324-5p in patients with GCa was evaluated by Kaplan-Meier survival curve and Cox regression analysis. Gain- and loss-of-function experiments were performed to evaluate the biological function of miR-324-5p during the progression of GCa, and a target gene of miR-324-5p was proposed.

**Results:**

The expression of miR-324-5p was up-regulated in GCa tissues and cell lines. Patients with high expression of miR-324-5p had more cases with positive lymph node metastasis, advanced TNM stage, and worse overall survival compared with patients with low expression. The elevated miR-324-5p was an independent prognostic indicator of GCa. In addition, the inhibition of miR-324-5p could suppress GCa cell proliferation, migration and invasion and promote cell apoptosis, and PTEN was demonstrated to serve as a direct target of miR-324-5p in GCa progression.

**Conclusion:**

The present study indicates that miR-324-5p overexpression predicts poor prognosis in GCa patients, and the reduction of miR-324-5p can inhibit GCa biological processes. PTEN is a target gene of GCa, which may mediate the biological function of miR-324-5p in GCa progression.

**Supplementary Information:**

The online version contains supplementary material available at 10.1186/s13000-020-01063-2.

## Introduction

Gastric cancer (GCa) is one of the six major malignancies worldwide, and its fatality rate is second only to lung cancer [1]. GCa mostly originates from chronic gastritis caused by Helicobacter pylori infection [2]. At present, GCa is mainly treated by surgery, supplemented by radiotherapy, chemotherapy and medication [3]. Although significant progress has been made in its diagnosis and treatment, most patients are diagnosed with advanced GCa or metastatic cancer cells due to atypical symptoms of early GCa and insufficient attention to early screening [4]. Therefore, improving the efficiency of early screening is of great significance for improving the overall survival rate of patients with GCa. Advances in endoscopic technology make it possible to detect GCa at an early stage, but the sensitivity is low. In recent years, the diagnosis and prognosis of GCa patients have improved significantly, but the prognosis of patients with advanced GCa is still not optimistic [5, 6]. The survival rate of GCa patients is less than 30% within 5 years. It is critical to reducing GCa mortality by developing and identifying new drug candidates.

MicroRNAs (miRNAs) are a series of endogenous non-coding small RNAs, about 21–23 nucleotides, which regulate gene expression by combining with complementary sequences in the target mRNA, resulting in its silencing or activation. MiRNA is involved in different cellular processes, such as metabolism, differentiation, development and apoptosis [7]. Studies have shown that miRNAs are abnormally expressed in malignant tumors and act as oncogenes or tumor suppressor genes during the development and metastasis of cancer [8, 9]. Some miRNA, such as miR-223-3p, miR-625-3p and miR-26a-5p, are key regulators of the development of GCa cells, and are involved in the proliferation, invasion, and metastasis of cancer cells [7, 10, 11]. These studies suggest that abnormally expressed miRNAs may be potential biomarkers for GCa. At the same time, the development of miRNA microarray chip analysis with cancer tissues and cells has provided us with miRNA markers that can be used as prognostic, diagnostic, and therapeutic tools to improve clinical efficacy [12]. At present, the diagnostic value of miR-324-5p in prostate cancer, rectal cancer, and lung cancer has been proved [13–15], but there is less research about the clinical value of miR-324-5p in GCa patients.

This study was aimed to investigate the expression of miR-324-5p in GCa, and to analyze its prognostic value and biological function. First, qRT-PCR was used to calculate the expression of miR-324-5p in tumor tissues and cell lines. The prognostic value of miR-324-5p in GCa was evaluated by Kaplan-Meier survival curve and Cox regression analysis. The regulatory effect of miR-324-5p on GCa cell proliferation, migration and invasion was further analyzed. Besides, the target gene of miR-324-5p that might mediate the function of miR-324-5p in GCa was predicted and assessed. This study is expected to obtain a novel biomarker and therapeutic target for GCa treatment.

## Materials and methods

### Patients and tissue collection

Totally 122 GCa tissue samples were collected at Weifang People’s Hospital from 2009 to 2013. Patients included 76 males and 46 females with an average age of 57.8 ± 23.2. All patients were pathologically diagnosed as GCa in the surgery and none of them had received any treatment before the operation. Patients met the following criteria were excluded from the analysis of this study: (1) cases received preoperative treatment; (2) cases had incomplete electronic medical records; (3) cases with immune system diseases; (4) cases with mental and cognitive dysfunction; (5) pregnant or lactation women; (6) cases died from other diseases or unexpected events. The personal information of patient is kept confidential and each patient has provided the written informed consent. Patients were followed up for a five-year investigation after surgery. The survival information and clinical pathological characteristics of patients with GCa was recorded. The study has been approved by the Ethics Committee of Weifang People’s Hospital.

### Cell culture and transfection

Four GCa cell lines, including AGS, HGC27, HS746T and MKN45, and one gastric epithelial cell line GES-1, which was used as a normal control, were purchased from the Type Culture Collection of the Chinese Academy of Sciences (Shanghai, China). The cells were cultured in RPMI-1640 medium (BioTek Corporation, Beijing, China) containing 10% fetal bovine serum (FBS; Thermo Fisher, Waltham, MA, USA) at 37 °C with 5% CO2. For cell transfection, AGS and MKN45 cells (density of 2 × 10^6^ cell/well) were seeded into 6-well plates and cultured to about 80% confluence, then were transfected with miR-324-5p mimics (50 nM), miR-324-5p inhibitors (100 nM) or negative controls, including 50 nM mimic NC and 100 nM inhibitor NC (GenePharma, Shanghai, China) using Lipofectamine 3000 (Invitrogen, Carlsbad, CA, USA) to regulate the expression of miR-324-5p in GCa. To overexpress the expression of PTEN in AGS cells, pcDNA3.1-PTEN was also transfected into cells using Lipofectamine 3000 (Invitrogen, Carlsbad, CA, USA) following the manufacturer’s instruction. The sequences were as follows: miR-324-5p mimic, 5′-CGCAUCCCCUAGGGCAUUGGUGU-3′; miR-324-5p inhibitor, 5′-ACACCAAUGCCCUAGGGGAUGCG-3′; mimic NC, 5′-UUCUCCGAACGUGUCACGU-3′; inhibitor NC, 5′-CAGUACUUUUGUGUAGUACAA-3′. The subsequent cell experiments were carried out at 48 h post-transfection.

### RNA extraction and Quantitative Real-Time PCR (qRT-PCR)

Total RNA was extracted from GCa tissues and cells by TRIzol reagent (Invitrogen, Carlsbad, CA, USA), and cDNA was obtained by reverse transcription with the reaction procedure of 42 °C for 30 min and 85 °C for 10 min using a PrimeScript RT reagent kit (TaKaRa, Shiga, Japan). SYBR-Green I Master Mix kit (Invitrogen, Carlsbad, CA, USA) and 7300 Real-Time PCR system (Applied Biosystems, USA) were used to perform the qPCR with the following thermocycling conditions: 95 °C for 10 min, followed by 40 cycles of 95 °C for 20 s, 60 °C for 10 s and 72 °C for 20 s. The obtained cDNA was a template for qPCR, and U6 and GAPDH was the internal reference gene. The melting curves for U6 and GAPDH could be observed in Supplementary Figure 1, which showed the single peak and the uniform Tm value for each internal reference. The relative expression of miR-324-5p and the mRNA levels of PTEN were calculated using the 2^-ΔΔCt^ method [16]. The sequences of primers were as follows: miR-324-5p forward 5′-GCCGAGCGCATCCCCTAGG-3′, reverse 5′-CTCAACTGGTGTCGTGGA-3′; PTEN forward 5′-AGTTCCCTCAGCCGTTACCT-3′, reverse ATTTGACGGCTCCTCTACTG-3′; U6 forward 5′-CTCGCTTCGGCAGCACA-3′, reverse 5′-AACGCTTCACGAATTTGCGT-3′; GAPDH forward 5′-CAATGACCCCTTCATTGACC-3′, reverse 5′-TTGATTTTGGAGGGATCTCG-3′.

### Cell proliferation assay

After 48 h of cell transfection, the tumor cells were seeded in 96-well plates with a cell density of 3 × 10^3^ per well. The cell plates were cultured in an incubator at 37 °C for 3 days, and 10 μl MTT was added at each well at different time points (24, 48 and 72 h). Then a volume of 150 μl DMSO was added in the wells. After 4 h of incubation, the optical density was measured at 570 nm on a microplate Reader.

### Cell apoptosis assay

GCa cell apoptosis was examined using an Annexin V-FITC Apoptosis Detection kit (Nanjing KeyGen Biotech, Nanjing, China). After 48 h of cell transfection, the cells were harvested and washed using PBS for three times, then were suspended with Annexin V binding buffer. The cells were stained using 5 μL V-FITC and 10 μL propidium iodide (PI) in the dark at room temperature for 20 min. Cell apoptosis was measured using a flow cytometer (BD Biosciences) and analyzed using the CellQuest Pro analysis software (BD Biosciences).

### Cell migration and invasion assay

For cell invasion assay, the Transwell chambers (Corning, USA) were precoated with 50 μL Matrigel (Corning, USA), which was diluted with serum-free RPMI-1640 medium (1:10). The Transwell chambers without Matrigel were used for migration assay. Tumor cells (density of 3 × 10^5^ cell/well) with serum-free culture medium were seeded into the upper chambers, and the lower chambers included medium containing 10% FBS. After 48 h incubation at 37 °C, the cells in the lower chambers were stained and the cell number in five random fields was counted under an inverted microscope (Olympus Corporation, Tokyo, Japan).

### Dual-luciferase reporter assay

According to the in silico prediction with miRanda (http://www.microrna.org/microrna/home.do), a complementary sequence of miR-324-5p was searched at the 3′-UTR of PTEN. To confirm the interaction between miR-324-5p and PTEN, a luciferase reporter assay was used. The wild type (WT) or mutant type (MT) 3′-UTR of PTEN was cloned into pGL3-luciferase basic vector (Promega, Madison, WI, USA). The combined vectors were co-transfected with miR-324-5p mimic, miR-324-5p inhibitor or the NCs into AGS cells by Lipofectamine 3000 (Invitrogen, Carlsbad, CA, USA) according to the protocols of manufacturers. After 24 h transfection, the relative luciferase activity was measured using a Dual-Luciferase Reporter Assay System (Promega).

### Statistical analysis

All the experiments and examinations were performed at least three times. Data in this study were analyzed using SPSS 21.0 and described as the mean ± SD. Comparisons between groups were analyzed by Student’s t test or one-way ANOVA. The relationship between miR-324-5p and clinicopathological data of GCa patients was performed using the χ^2^ test. Kaplan-Meier survival and Cox regression analysis were adopted to examine the prognostic value of miR-324-5p. *P*< 0.05 was considered to indicate a statistically significant difference.

## Results

### Upregulation of miR-324-5p in GCa tissues and cell lines

**Fig. 1:**
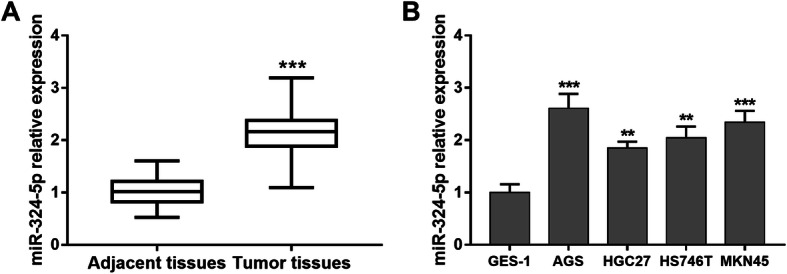
Expression of miR-324-5p in GCa patients and cell lines. **a**. Tumor tissues had higher miR-324-5p expression than the adjacent normal tissues (***P < 0.001). **b**. Expression of miR-324-5p was elevated in GCa cell lines compared with the normal cell line (**P < 0.01, ***P < 0.001)

The expression of miR-324-5p in GCa tissues and cell lines was detected by qRT-PCR. As shown in Fig. 1a, the expression of miR-324-5p in GCa patients was up-regulated compared with adjacent normal controls (*P* < 0.001). In addition, the expression of miR-324-5p in GCa cell lines was also higher than that in gastric epithelial cells (*P* < 0.01, Fig. 1b).

### Association of miR-324-5p with clinicopathological characteristics of GCa patients

**Fig. 2:**
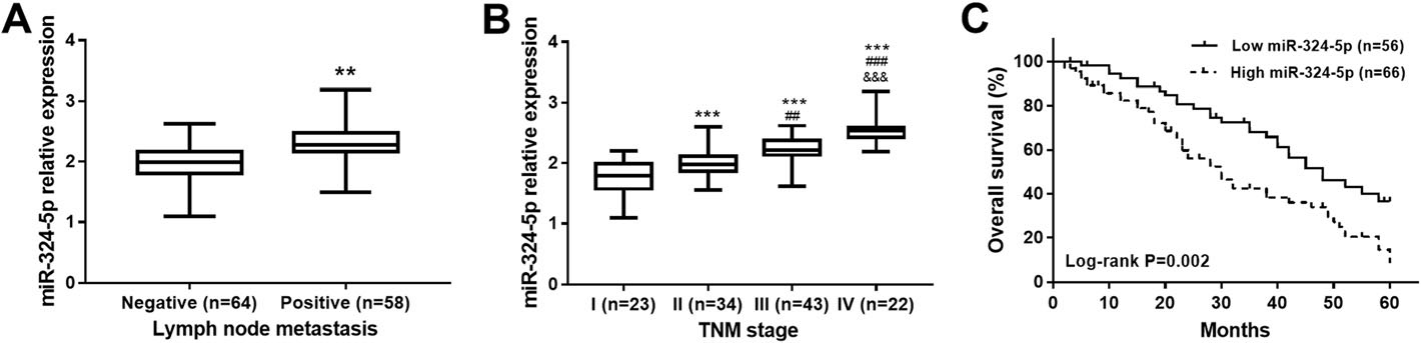
Association of miR-324-5p with lymph node metastasis, TNM stage and overall survival in patients with GCa. **a**. GCa patients with positive lymph node metastasis had significant high miR-324-5p expression (**P < 0.01). **b**. miR-324-5p expression was elevated with the increase of TNM stage, and the highest miR-324-5p expression was observed in patients with TNM stage IV (***P < 0.001 compared to stage I, ^##^*P* < 0.01, ^###^*P* < 0.001 compared to stage II, ^&&&^*P* < 0.001 compared to stage III). **c**. Survival curves for GCa patients based on Kaplan-Meier method (log-rank *P* = 0.002)

We found that miR-324-5p may be involved in the development of GCa by analyzing the association between miR-324-5p and clinical data of GCa patients. The mean expression value of miR-324-5p (2.127) was used as the cutoff value, patients were divided into low and high expression groups in order to analyze the relationship between miR-324-5p and clinical pathological data of patients. The results listed in Table 1 showed that the expression of miR-324-5p was related to lymph node metastasis and TNM stage (all *P* < 0.05). However, there was no association between miR-324-5p and other parameters, including age, gender, tumor size and differentiation (all *P*> 0.05). Furthermore, the expression of miR-324-5p in GCa patients with different status of lymph node metastasis and TNM stages was compared. As expected, the expression of miR-324-5p was significantly increased in the patients with positive lymph node metastasis and advanced TNM stages (all *P* < 0.01, Fig. 2a and b). miR-324-5p expression was elevated with the increase of TNM stage, and the highest miR-324-5p expression was observed in patients with TNM stage IV (all *P* < 0.01).

**Table 1:** Relationship between miR-324-5p and clinicopathological characteristics of gastric cancer patients

Features	Category	All (*n*=122)	miR-324-5p expression		*P*
			Low (*n*=56)	High (*n*=66)	
Age (years)	< 60	49	22	27	0.855
	≥ 60	73	34	39	
Gender	Female	46	21	25	0.966
	Male	76	35	41	
Tumor size (cm)	< 5	58	30	28	0.219
	≥ 5	64	26	38	
Differentiation	Well-moderate	65	35	30	0.060
	Poor	57	21	36	
Lymph node metastasis	Negative	64	37	27	0.006
	Positive	58	19	39	
TNM stage	I	23	17	6	0.002
	II	34	17	17	
	III	43	18	25	
	IV	22	4	18

### Clinical significance of miR-324-5p in the prognosis of GCa

Kaplan-Meier survival curve and Cox regression analysis were used to assess the prognostic value of miR-324-5p. As shown in Fig. 2c, patients with low expression of miR-324-5p had higher overall survival compared to patients with high expression of miR-324-5p (log-rank *P* = 0.002). Furthermore, the multivariate Cox analysis results revealed that the expression of miR-324-5p served as an independent prognostic biomarker in patients with GCa (HR = 1.791, 95% CI = 1.070–2.999, *P* =0.027) (Table 2).

**Table 2:** Cox regression analysis for the overall survival of patients with gastric cancer

Indicators	Hazard ratio	95% CI	*P*
miR-324-5p	1.791	1.070–2.999	0.027
Age	1.356	0.843–2.181	0.209
Gender	1.161	0.704–1.915	0.558
Tumor size	1.330	0.803–2.202	0.268
Differentiation	1.449	0.806–2.389	0.128
Lymph node metastasis	1.528	0.926–2.521	0.097
TNM stage	1.514	1.034–2.522	0.038

### miR-324-5pregulates GC cell proliferation, apoptosis, migration and invasion

**Fig. 3:**
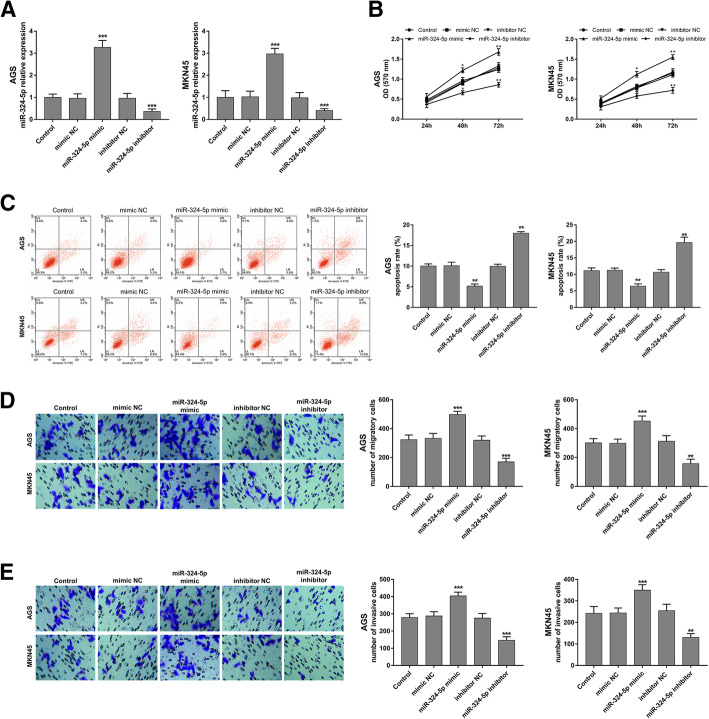
Effect of miR-324-5p on cell proliferation, apoptosis, migration and invasion in AGS and MKN45 cell lines. **a**. miR-324-5p expression was upregulated by the miR-324-5p mimic and downregulated by the miR-324-5p inhibitor in GCa cells. **b**. The overexpression of miR-324-5p enhanced cell proliferation, but the silencing of miR-324-5p inhibited cell proliferation in both the AGS and MKN45 cell lines. **c**. miR-324-5p overexpression led to reduced cell apoptosis, but miR-324-5p reduction resulted in increased cell apoptosis in both AGS and MKN45 cell lines. **d**. The migration ability of both two GCa cell lines was promoted by the overexpression of miR-324-5p, but was suppressed by the knockdown of miR-324-5p. **e**. The invasion of AGS and MKN45 cells was accelerated by the upregulation of miR-324-5p while inhibited by the downregulation of miR-324-5p. *P < 0.05, **P < 0.01, ***P < 0.001

This study analyzed the regulatory effects of miR-324-5p on the proliferation, apoptosis, migration and invasion of both AGS and MKN45 cells. After cell transfection, the expression of miR-324-5p in both AGS and MKN45 cell lines was significantly increased by the miR-324-5p mimic, and was reduced by the miR-324-5p inhibitor (all *P* < 0.001, Fig. 3a). For cell proliferation, the overexpression of miR-324-5p could markedly promote AGS and MKN45 cell proliferation, while the reduction of miR-324-5p could inhibit the proliferation of AGS and MKN45 cells (all *P* < 0.05, Fig. 3b). The cell apoptosis results showed that the overexpression of miR-324-5p inhibited cell apoptosis rate, while the inhibition of miR-324-5p led to enhanced cell apoptosis rate in both AGS and MKN45 cell lines (all *P* < 0.01, Fig. 3c). Transwell assays were performed to assess the influence of miR-324-5p on GCa cell migration and invasion. As presented in Fig. 3d and e, compared with the untreated cells, miR-324-5p overexpression induced GCa cells migration and invasion, and a downregulation of miR-324-5p inhibited GCa cells migration and invasion (all *P* < 0.01).

### PTEN directly mediates the regulatory effects of miR-324-5p on GCa cell biological function

**Fig. 4:**
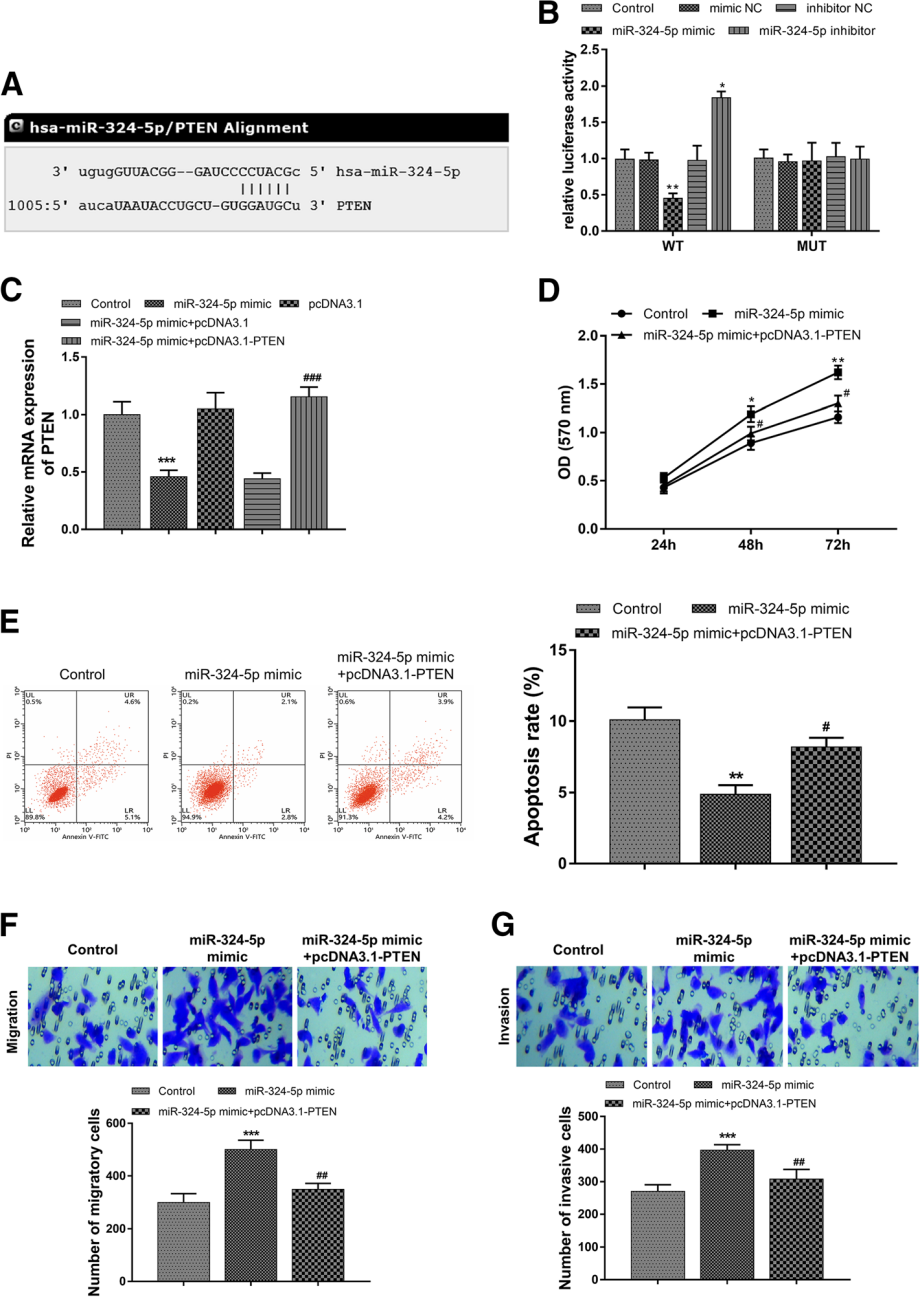
PTEN directly mediated the regulatory effects of miR-324-5p on AGS cell proliferation, apoptosis, migration and invasion. **a**. The putative binding site of miR-324-5p at the 3′-UTR of PTEN. **b**. The relative luciferase activity in AGS cells in the dual-luciferase reporter assay. **c**. The mRNA expression of PTEN was inhibited by miR-324-5p overexpression, and the pcDNA3.1-PTEN could reverse this effect. **d-g**. The promoted cell proliferation, inhibited cell apoptosis and enhanced cell migration and invasion induced by miR-324-5p overexpression were all abolished by the upregulation of PTEN in AGS cells. *P < 0.05, **P < 0.01, ***P < 0.001 compared to control group; ^#^*P* < 0.05, ^##^*P* < 0.01 compared to miR-324-5p mimic group

A putative binding site of miR-324-5p was found at the 3′-UTR of PTEN (Fig. 4a). Consequently, a dual-luciferase reporter assay was conducted to confirm the interaction between miR-324-5p and PTEN. As shown in Fig. 4b, the relative luciferase activity in AGS cells transfected with WT vectors was significantly inhibited by the overexpression of miR-324-5p, but was enhanced by the reduction of miR-324-5p (all *P* < 0.05). In contrast, no significant changes were observed in the MUT group for the relative luciferase activity (*P* > 0.05). In addition, the relative mRNA expression of PTEN was significantly inhibited by the overexpression of miR-324-5p (*P* < 0.001, Fig. 4c). These findings indicated that PTEN might be a direct target of miR-324-5p in GCa cells. Furthermore, the reduced PTEN expression caused by miR-324-5p overexpression was recovered in AGS cells after co-transfected with pcDNA3.1-PTEN (*P* < 0.001, Fig. 4c), and we found that the enhanced proliferation, reduced cell apoptosis and increased cell migration and invasion of AGS cells induced by miR-324-5p overexpression were all reversed by the increased expression of PTEN (all *P* < 0.05, Fig. 4d-g).

## Discussion

The rapid development of molecular biology and modern medicine proves that miRNAs are closely related to various diseases such as cardiovascular disease and cancer. For example, overexpression of miR-324-5p-3p can regulate multiple genes associated with cardiac function, such as cellular stress, metabolism, cell survival and apoptosis [17]. MiRNAs such as miR-155, miR-21 and miR-221 are abnormally expressed in rectal and colon cancer, but the expression of miRNA are different between the two groups [18]. Therefore, exploring new functional miRNAs is of great value for improving cancer treatment. Further, studies show that there are a large number of abnormally expressed miRNA in GCa, such as miR-711 [19], miR-146a [20], miR-140-5p [21]. These abnormally miRNAs in GCa may be promising biomarkers for GCa diagnosis. Clinically effective prognostic biomarkers can not only indicate the progression and metastasis of potential cancers, but also can help clinicians to develop more appropriate treatment strategies for cancer patients. Therefore, identifying more potential functional miRNAs is of great important for the treatment of GCa.

In this study, we explored a novel miRNA—miR-324-5p, which is associated with GCa. A large number of studies have shown that miR-324-5p is closely related to various cancers [22]. Jin et al. explored the value of miR-324-5p in the early diagnosis of prostate cancer [23]. Bamodu et al. found that the enhanced expression of miR-324-5p-5p in colorectal cancer cells inhibited its tumorigenicity in vitro and vivo [24]. Our study also confirmed that miR-324-5p expression was significantly higher in GCa tissues and cell lines than the controls, and the overexpression of miR-324-5p predicted a poor prognosis for GCa patients, which is consistent with the research by Shrestha [25]. Correlation analysis between miR-324-5p and clinical data of GCa patients showed that miR-324-5p was associated with tumor grade and poor prognosis. These experimental data indicate that miR-324-5p may be a potential oncogene in GCa and participate in the occurrence and development of GCa. Cancer cachexia is closely associated with increased cancer mortality, especially in GCa [26]. However, this study did not evaluate the weight loss and nutritional status of the enrolled GCa patients, and thus could not analyze the relationship of miR-324-5p with cachexia onset and cachexia-related GCa mortality. This is one of the limitations of this study, and further investigations should focus on the nutritional status and development of cachexia.

Based on hundreds of thousands of expression analysis studies, it has been clearly known that tumors generally show uncontrolled miRNA expression patterns relative to normal tissues. The expression pattern of miRNA generally shows uncontrol in tumors. miRNA expression patterns provide useful information for tumor classification and prediction [27]. miRNAs are involved in the regulation of the cell cycle, apoptosis and cell invasion, which indicates their potential as diagnostic and therapeutic markers to enhance prognosis and heighten cancer survival [28, 29]. Given the abnormal expression of miR-324-5p in GCa, this study analyzed the clinical significance of its diagnosis and prognosis in GCa. The prognostic value of miR-324-5p was evaluated based on the 5-year survival information of GCa patients. Kaplan-Meier survival curves showed that patients with high expression of miR-324-5p had worse overall survival compared with those patients with low miR-324-5p levels. In addition, miR-324-5p is independently related to overall survival. These studies suggest that miR-324-5p may be a potential prognostic biomarker for GCa.

Studies have shown that the growth, proliferation, invasion, metastasis, apoptosis and tumor angiogenesis of malignant tumor cells are closely related to abnormal miRNA [28]. The cell experiments in this study showed that the overexpression of miR-324-5p could promote GCa cell proliferation, migration and invasion but inhibit cell apoptosis. The regulatory effects of miR-324-5p on cell biological function have been previously reported in colorectal cancer and lung cancer [30, 31], indicating the functional role of miR-324-5p in human malignancies. Thus, we considered that miR-324-5p might play an oncogenic role in the progression of GCa. However, the effects of miR-324-5p on GCa carcinogenesis need to be confirmed by in vivo experiments in future studies. The regulatory effects of miR-324-5p on GCa cell migration and invasion led us to wonder if there was also a link between miR-324-5p and GCa cell motility, owing to that motility is an important process for cancer metastasis [32]. Thus, further investigations using cell-tracking assay may interpret this deduction and enrich the functional role of miR-324-5p in GCa progression. In addition, the mechanism of action of miR-324-5p in GCa is not clear. Studies have shown that hypermethylation of the promoter CpG island region in GCa leads to epigenetic silencing of miRNAs [29]. Based on existing research and conclusions, we believe that miR-324-5p may regulate the proliferation or metastasis of GCa cells by binding to the 3′-UTR region of the target gene to activate or inactivate related signaling pathways.

PTEN is a widely known as a tumor suppressor, and its mutation or downregulation has been commonly observed in various human cancers, including GCa [33, 34]. The most important function of PTEN is acting as an inhibitor of the PI3K/AKT signaling pathway, which is a pivotal signal during cancer cell growth [35]. Numerous key molecules are involved in GCa progression through the PI3K/AKT signaling pathway [36, 37]. In this study, we found a putative binding site of miR-324-5p at the 3′-UTR of PTEN. Furthermore, the dual-luciferase reporter assay results demonstrated that miR-324-5p could directly bind the 3′-UTR of PTEN in GCa cells, and the regulatory effects of miR-324-5p overexpression on AGC cell proliferation, apoptosis, migration and invasion were reversed by PTEN upregulation. Thus, we considered that the biological function of miR-324-5p in GCa progression might be achieved through the PTEN/PI3K/AKT pathway. However, the activity of PI3K/AKT signaling was not examined in this study. To further uncover the mechanisms underlying the functional role of miR-324-5p, the expression of PTEN and the proteins involving in PI3K/AKT signaling need to be examined in GCa tissues and cell lines in future studies. In addition, this study investigated the role of miR-324-5p in GCa only by in vitro experiments, further studies should confirm the function of miR-324-5p using in vivo experiments.

In summary, the present results demonstrated that miR-324-5p expression is up-regulated in the tissues and cells of GCa, and its high expression is associated with advanced TNM tumor stage and predicts a poor prognosis for patients with GCa. In addition, the reduction of miR-324-5p can inhibit cell proliferation, migration and invasion of GCa cells, which indicates the potential oncogenic role of miR-324-5p in GCa progression. Overall, all the findings in this study may provide a novel non-invasive biomarker to predict overall survival outcomes for patients with GCa, and the methods to inhibit miR-324-5p may provide a novel insight into the development of therapeutic approaches for GCa.

## Supplementary Information

**Additional file 1: Figure S1.** Melting curves of U6 (A) and GAPDH (B).

## Data Availability

All data generated or analyzed during this study are included in this published article.

## Abbreviations

GCa: Gastric cancer
miRNAs: microRNAs
NC: Negative control
qRT-PCR: Quantitative real-time PCR
