# Supplementary figures and images for: High miR-324-5p expression predicts unfavorable prognosis of gastric cancer and facilitates tumor progression in tumor cells
Source: Diagn Pathol. 2021 Jan 11;16:5. doi: 10.1186/s13000-020-01063-2 (PMC7798222; doi:10.1186/s13000-020-01063-2)

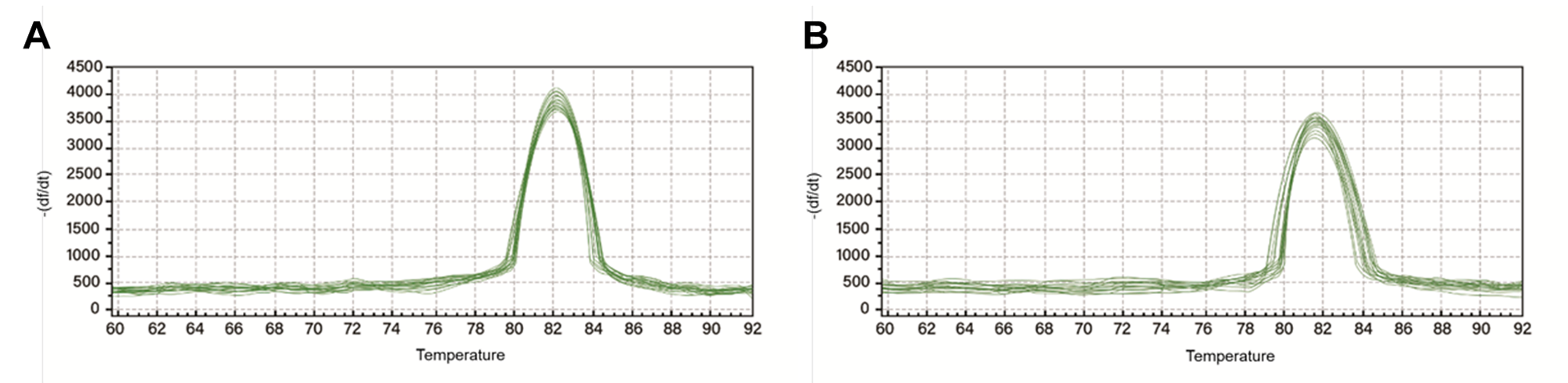

Supplement: Supplementary file 1 — Additional file 1: Figure S1. Melting curves of U6 (A) and GAPDH (B). [file 13000_2020_1063_MOESM1_ESM.tif]
